# Impact of Sarcopenia on Non-Alcoholic Fatty Liver Disease

**DOI:** 10.3390/nu15040891

**Published:** 2023-02-10

**Authors:** Michihiro Iwaki, Takashi Kobayashi, Asako Nogami, Satoru Saito, Atsushi Nakajima, Masato Yoneda

**Affiliations:** Department of Gastroenterology and Hepatology, Yokohama City University School of Medicine, Graduate School of Medicine, 3-9 Fukuura, Kanazawa-ku, Yokohama 236-0004, Japan

**Keywords:** non-alcoholic fatty liver disease 1, sarcopenia 2, non-alcoholic steatohepatitis 3, sarcopenia-associated obesity 4

## Abstract

With the increasing incidence of non-alcoholic fatty liver disease (NAFLD) and the aging of the population, sarcopenia is attracting attention as one of the pathological conditions involved in the development and progression of NAFLD. In NAFLD, sarcopenia is closely associated with insulin resistance and results from the atrophy of skeletal muscle, an insulin target organ. In addition, inflammatory cytokines that promote skeletal muscle protein breakdown, low adiponectin levels leading to decreased insulin sensitivity, and hyperleptinemia are also involved in NAFLD pathogenesis. The presence of sarcopenia is a prognostic factor and increases the risk of mortality in patients with cirrhosis and post-treatment liver cancer. Sarcopenia, the presence of which mainly occurs due to decreased muscle mass, combined with increased visceral fat, can lead to sarcopenia-associated obesity, which increases the risk of NASH, liver fibrosis, and cardiovascular disease. In order to treat sarcopenia, it is necessary to properly evaluate sarcopenia status. Patients with high BMI, as in sarcopenic obesity, may improve with caloric restriction. However, inadequate oral intake may lead to further loss of muscle mass. Aerobic and resistance exercise should also be used appropriately.

## 1. Introduction

Non-alcoholic fatty liver disease (NAFLD) and non-alcoholic steatohepatitis (NASH) are among the most common liver diseases, with the number of patients increasing rapidly worldwide due to the increasing prevalence of obesity and type 2 diabetes. NASH is considered to be the most common liver disease [1] and is recognized as a chronic progressive disease that increases the risk of various complications such as chronic liver disease, obesity, cancer, and cardiometabolic diseases such as T2DM [2]. With the increase in the number of patients with NAFLD, sarcopenia has attracted attention as one of the pathological conditions involved in the development and progression of NAFLD. Sarcopenia was first reported in 1989 as a condition characterized by age-related loss of muscle mass [3], and in 2010, the European Working Group on Sarcopenia in Older People published a diagnostic criteria [4], making sarcopenia a widely recognized disease. For many years, sarcopenia was thought to occur exclusively in the geriatric population; however, it is now recognized that sarcopenia can occur early in life [5]. In recent years, several studies have shown a correlation between sarcopenia and chronic diseases such as type 2 diabetes (T2DM) [6], metabolic syndrome (MetS) [7], and liver disease [8]. Sarcopenia has been reported to be associated with increased morbidity and mortality, worse quality of life, and disability [9]. This article outlines the current status of NASH/NAFLD and the clinical significance of sarcopenia and its pathogenesis in NASH/NAFLD.

## 2. Epidemiology of NAFLD/NASH

NAFLD is classified into nonalcoholic fatty liver (NAFL) with slow progression to liver fibrosis and NASH with hepatocellular damage and liver fibrosis. According to a meta-analysis, the prevalence of NAFLD is 25.2% worldwide, 27.4% in Asia [10], and 22.3% in Japan [11]. Projection models have predicted that by 2030, the number of NAFLD cases would increase by 18.3% and reach 109 million, with a prevalence rate of 28.4% [12]. According to the model, the number of patients with NAFLD is expected to increase significantly in China and the United States by 2030, while this number is expected to level off in Japan, partly due to population decline. The proportion of patients with NASH in the NAFLD population will also increase in the coming decades due to an aging population and the projected increase in the prevalence of DM among older adults. Approximately 20% of NAFLD cases were classified as NASH in 2015 and this rate is expected to reach 27% by 2030. The incidence of non-compensated cirrhosis will increase by 168% from 39,320 cases to 105,430 cases by 2030. Similarly, the incidence of hepatocellular carcinoma will increase by 137% from 5160 to 12,240 cases by 2030, and liver deaths are predicted to increase by 178% from 28,200 to an estimated 78,300 by 2030.

In Japan, the proportion of cases with advanced liver fibrosis is expected to increase as the population ages [12], and similarly, the number of cases with sarcopenia complications is expected to increase.

## 3. Sarcopenia in Chronic Liver Disease

Skeletal muscle as a metabolic organ as well as a locomotor organ is increasingly attracting researchers’ and physicians’ attention. Sarcopenia is defined as an age-related disease involving a decrease in muscle quantity and quality as well as physical performance [3,13]. Specifically, sarcopenia is probable when low muscle strength is detected. A sarcopenia diagnosis is confirmed by the presence of low muscle quantity or quality. When low muscle strength, low muscle quantity/quality and low physical performance are all detected, sarcopenia is considered severe [3]. Primary sarcopenia is caused solely by aging, secondary sarcopenia is defined as sarcopenia caused by various factors such as liver disease. The three types of secondary sarcopenia include those related to chronic disease, those related to inactivity, and those associated with nutrition. There is a close relationship between skeletal muscle and liver, in particular, in terms of glucose, amino acid, and ammonia metabolism [14].

Nutritional disorders, low levels of branched-chain amino acids (BCAAs), hyperammonemia, abnormal gut microbiota, insulin resistance, and lipid factors are thought to contribute to sarcopenia in patients with chronic liver disease [15]. Cirrhosis, a terminal manifestation of chronic liver disease, often results in protein-energy malnutrition (PEM). When protein hyponutrition was evaluated by albumin level (<3.5 g/dL) and energy hyponutrition by respiratory quotient (<0.85) in indirect calorimetry, 48% of patients with cirrhosis were reported to be energy hyponutrient and 67% were protein hyponutrient, with 18% of PEM patients having both [16]. Although glucose is an important energy-producing substance, amino acids are used for glycogenesis due to low glycogen stores in the liver and the unavailability of fatty acid carbons for glycogenesis in patients with chronic liver disease. Amino acids are mainly supplied by the breakdown of skeletal muscle, in which BCAAs are mainly degraded and used for glycogenesis [17]. Because BCAAs activate the mechanistic/mammalian target of rapamycin complex 1 (mTORC1) and induces protein anabolism in muscle cells [18], sarcopenia is more likely to progress with decreased blood BCAAs in chronic liver disease. Hyperammonemia may contribute to decreased muscle protein synthesis by interfering with the tricarboxylic acid (TCA) cycle [19,20]. Hyperammonemia is also associated with increased reactive oxygen species and may lead to muscle loss [19]. Deterioration of the intestinal environment leads to an increase in anaerobic Gram-negative rods and an increase in lipopolysaccharide (LPS) in the blood, which is a component of the outer wall membrane of Gram-negative rods and a positive endotoxin [21]. Patients with cirrhosis show an increased LPS concentration in the portal vein in proportion to the degree of progression [22,23]. Endotoxin is a member of the pathogen-associated molecular patterns (PAMPs), which comprise a group of receptors such as Toll-like receptors (TLRs) and nucleotide-binding oligomerization domain receptors (NLRs). In particular, TLR4 is expressed on the plasma membrane of hepatocytes and Kupffer cells, and TLR4-mediated signals are thought to activate signaling molecules such as NF-kB, leading to the production of inflammatory cytokines (IL-1b and IL-18) and the induction of liver damage [24]. Furthermore, for patients with cirrhosis, dysbiosis of intestinal bacteria causes hyperammonemia and is considered to be involved in sarcopenia and insulin resistance [25]. It has also been suggested that amino acids synthesized by the microbiome are associated with sarcopenia [26]. Insulin resistance is largely related to sarcopenia and liver diseases including NAFLD (described in the Insulin Resistance in Sarcopenia of NAFLD/NASH section). Lipid factors are related to the release of inflammatory cytokines (described in the Hormonal and Cytokine Changes in Sarcopenia in NAFLD/NASH section).

It has been reported that sarcopenia is associated with cirrhosis that has progressed to liver failure in approximately 60% of patients and is a prognostic determinant in cirrhosis [14,27,28]. Sarcopenia is also an independent predictor of mortality in cirrhosis and is associated with a higher prevalence of portal hypertension, higher infection rates, longer hospital stays, hepatocellular carcinoma, and worse outcomes after liver transplantation [29]. In one meta-analysis, sarcopenia was associated with 48.1% of all cirrhosis cases. In terms of prognosis, survival was significantly worse in the sarcopenia group, with more cases dying due to infection [30]. High mortality rates have been reported in cirrhotic patients with sarcopenia when the death is associated with sepsis [31]. These studies suggest that the increased risk of sepsis is the primary reason contributing to mortality in cirrhosis with sarcopenia.

## 4. Insulin Resistance in Sarcopenia of NAFLD/NASH

**Figure 1:**
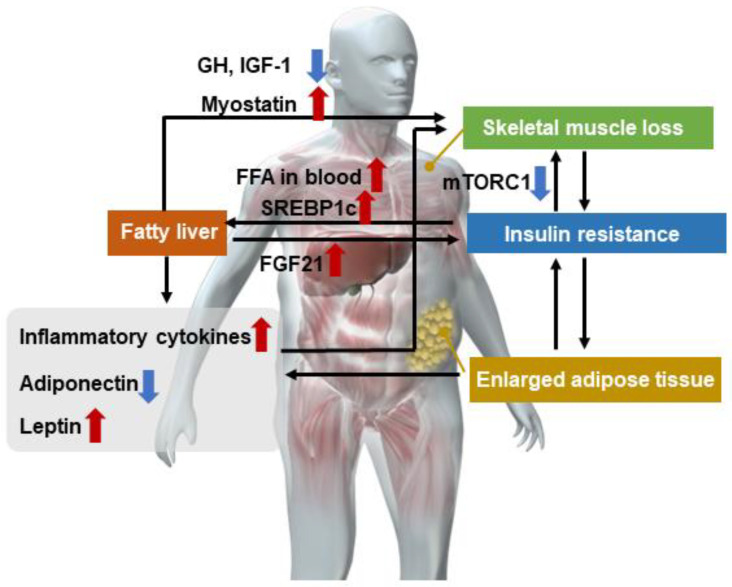
FFA, free fatty acid; FGF21, fibroblast growth factor 21; GF, growth factor; IGF-1, insulin-like growth factor: mTORC1, mechanistic/mammalian target of rapamycin complex 1; SREBP1c, Sterol regulatory element binding protein-1c. Upward arrows indicate enhancement and downward arrows indicate inhibition.

Skeletal muscle plays a major role in glucose transport and processing, fatty acid oxidation, and energy homeostasis, all being key determinants in the pathophysiology of NAFLD [32,33,34]. Orally ingested proteins are broken down into amino acids and peptides by the action of digestive juices (especially gastric and pancreatic juices) and absorbed via the small intestine. The absorbed amino acids are transported via the portal vein to the liver for the synthesis of various proteins and are then stored throughout the body [35,36,37,38]. The skeletal muscle is the largest amino acid storage organ in the body and plays an important role in glucose metabolism and fat deposition in the liver. Sarcopenia characterized by skeletal muscle loss, NAFLD, and abnormal glucose metabolism such as insulin resistance are closely related to each other (Figure 1). The progression of insulin resistance is considered to be a factor in the development of NAFLD and especially NASH [39,40,41]. Insulin resistance increases lipolysis in adipose tissue, leading to an increase in free fatty acids in the blood and FFA influx to the liver [42]. Sterol regulatory element binding protein-1c (SREBP-1c) is also activated when insulin resistance induces compensatory hyperinsulinemia. The activation of SREBP-1c results in increased fatty acid synthesis, and the excess fatty acids accumulate in the liver in the form of triglycerides, thereby contributing to the formation of fatty liver [43,44]. However, NAFLD could exacerbate insulin resistance by the following mechanisms. Hepatokines (cytokines secreted by the liver) such as Fetuin-A and fibroblast growth factor (FGF)-21 that are secreted due to endoplasmic reticulum stress in NAFLD/NASH were found to increase insulin resistance [45]. 

It has been reported that patients with NAFLD/NASH are prone to sarcopenia despite their high BMI, and the complication rate of sarcopenia ranges from 20.8% to 43.6%, with the complication rate increasing as fibrosis progresses [29,46]. Whether NAFLD contributes directly to sarcopenia remains controversial.

It has been suggested that the pathogenesis of sarcopenia is closely related to the development of insulin resistance. Sarcopenia and insulin resistance have reciprocal effects as described here. Sarcopenia promotes insulin resistance independent of obesity, since skeletal muscle is the major tissue involved in insulin-mediated glucose disposal [47,48]. However, it has been reported that insulin resistance promotes the progression of sarcopenia through the following mechanisms. In skeletal muscle, mTORC1 signaling has an important role. Hyperinsulinemia activates mTORC1, but prolonged mTORC1 activity causes negative feedback with insulin signaling, leading to decreased mTORC1 signaling [49]. mTORC1 is also a negative regulator of autophagy, and suppressor of mTORC1 Inhibition of mTORC1 causes accelerated autophagy and increased protein disassembly [50]. Chronic hyperinsulinemia results in the exacerbation of the pathogenesis of sarcopenia.

## 5. Hormonal and Cytokine Changes in Sarcopenia in NAFLD/NASH

In NAFLD/NASH, adipose tissue macrophages secrete inflammatory cytokines such as tumor necrosis factor-α (TNF-α), transforming growth factor-β (TGF-β), and interleukin-1 and -6 [2,51,52,53]. These cytokines promote protein decay in skeletal muscle [2,51,54]. In addition, growth hormone (GH) and insulin growth factor-1 (IGF-1) may be decreased in NAFLD/NASH, which may contribute to the progression of sarcopenia, since IGF-1 produced in the liver has a muscle-retaining effect [55]. 

In NAFLD/NASH, adiponectin is decreased and hyperleptinemia is observed. Adiponectin is a protein secreted exclusively from adipose tissue and is negatively correlated with fat accumulation. Adiponectin promotes insulin sensitivity by enhancing glucose uptake in skeletal muscle and adipose tissue and increases fatty acid oxidation [54,56]. In addition, adiponectin exerts anti-inflammatory effects and plays a hepatoprotective role in liver inflammation and cell damage [57,58,59,60]. It also improves mitochondrial function and insulin resistance in skeletal muscle [61,62]. 

Leptin stimulates fat oxidation in skeletal muscle. Hyperleptinemia due to leptin resistance is a condition in which leptin is less effective even when leptin levels are high. Hyperleptinemia is positively correlated with fat mass (FM) [63] and may promote insulin resistance, liver inflammation, and fibrosis [64]. Myostatin, which causes skeletal muscle atrophy, is a member of the TGF-α superfamily of glycoproteins and is produced by skeletal muscle. Myostatin enhances proteolysis via autophagy. Autophagy-mediated proteolysis is reported to be enhanced in muscle during cirrhosis and hyperammonemia [65]. It has been reported that blood myostatin levels increase in cirrhosis especially in the presence of a portal vein major circulation shunt [66], and is a factor in the pathogenesis of sarcopenia. Cirrhotic patients with high serum myostatin levels have a significantly lower cumulative survival rate than those with low serum myostatin levels [67]. It has also been reported that the hepatokine selenoprotein P, which is frequently expressed in type 2 diabetes, fatty liver, and elderly patients, acts on skeletal muscle and causes “exercise resistance” that nullifies the effects of exercise [68]. 

## 6. Prevalence and Clinical Significance of Sarcopenia in NASH/NAFLD

In recent years, clinical data have reported the coexistence of NASH/NAFLD and sarcopenia (Table 1). It is reported that the risk of NAFLD is significantly higher in patients with sarcopenia, regardless of the presence of obesity or metabolic syndrome [46], and that the presence of sarcopenia increases the risk of NAFLD more than fivefold [69]. The presence of sarcopenia has been reported to increase the risk of not only NAFLD but also fibrosis development with a risk ratio of 2.05 times [70]. Furthermore, sarcopenia increases mortality in patients with NAFLD [71,72,73]. Conversely, subjects with NAFLD have been reported to have significantly lower skeletal muscle index (SMI) compared with controls [74]. This finding was supported by a subsequent meta-analysis suggesting a direct relationship between sarcopenia and NAFLD [75]. However, it is difficult to establish a causal relationship. Cross-sectional studies of Asians have all reported that patients with NAFLD/NASH have a higher complication rate of sarcopenia, which also contributes to liver fibrosis [2,30,45,70], but the diagnostic methods for sarcopenia may differ, contributing to the difficulty in understanding the pathogenesis. 

Baumgartner proposed sarcopenia obesity as a condition caused by the combination of sarcopenia, which mainly comprises a decrease in muscle mass, and obesity, which comprises an increase in visceral fat [76]. It has been estimated that one in ten elderly people were diagnosed with sarcopenia obesity [77]. Shida et al. used the skeletal muscle mass-to-visceral fat area ratio (SVR) as an indicator of sarcopenic obesity and found that the SVR was strongly associated with liver stiffness and liver fibrosis markers, such as M2BPGi, as well as insulin resistance [78]. Other reports also support the association of sarcopenic obesity with NASH and liver fibrosis [70,79]. Other studies have reported that sarcopenia combined with obesity can increase the risk of cardiovascular diseases (CVD) such as type 2 diabetes, hyperlipidemia, and hypertension [80]. Sarcopenic obesity is also associated with higher morbidity and mortality than both sarcopenia alone and obesity alone [81]. Sarcopenia obesity was associated with multiple morbidities including CVD events (heart diseases and stroke), metabolic disorders, cognitive impairment, arthritis, physical disability, and lung diseases [82].

**Table 1:** Previous reports on the association between sarcopenia and nonalcoholic fatty liver disease.

Study and Year	Study Design, Sample Size, Population	Diagnosis of NAFLD	Diagnosis of Sarcopenia	Main Findings
Yong-ho Lee et al., 2015 [83]	Retrospective cohort 2761 subjects in Republic of Korea	NAFLD liver fat score	DEXA method	The risk of progression was significantly higher in patients with sarcopenia on NAFLD.
Ho Cheol Hong et al., 2014 [69]	Prospective observational cohort 452 subjects in Republic of Korea	The liver attenuation index on CT scan.	DEXA method	Individuals with lower muscle mass exhibited increased risk of NAFLD.
Koo, et al. [70]	Cross-sectional cohort (prospectively enrolled) 309 subjects in Republic of Korea	Liver biopsy	BIA method	Sarcopenia was significantly associated with NASH and significant fibrosis.
Golabi, et al. [71]	Retrospective cohort 1351 subjects in USA	U.S. Fatty Liver Index	DEXA method	Compared with NAFLD without sarcopenia, NAFLD with sarcopenia was associated with a higher risk of mortality.
Kim, et al. [72]	Retrospective cohort 11,065 subjects in USA	Ultrasonography	BIA method	Only in individuals with NAFLD, sarcopenia was associated with a higher risk for all-cause mortality, while this association was absent in those without NAFLD.

BIA, bioelectrical impedance analysis; CT, computed tomography; DEXA, dual energy X-ray absorptiometry; NAFLD, nonalcoholic fatty liver disease; NASH, nonalcoholic steatohepatitis.

## 7. Evaluation of Sarcopenia in NASH/NAFLD

A variety of tests and tools are available to assess sarcopenia. The European consensus on definition and diagnosis (EWGSOP2) recommends the SARC-F questionnaire to discover cases of sarcopenia [3]. The SARC-F includes the following five components: strength, walking with assistance, rising from a chair, climbing stairs, and falling [84]. Another study reported that the specificity of the test is very high [85]. 

This section describes the methods of assessing muscle strength, muscle mass and quantity, and physical performance, which define sarcopenia. The most useful methods of assessing muscle strength are grip strength measurements and chair stand tests. The grip strength test is very simple, and it is possible to measure grip strength in a wide range of practice settings. Poor grip strength is a strong predictor of a poor prognosis [86,87]. Among a cohort of NAFLD patients, low grip strength was independently associated with long-term all-cause mortality, suggesting that grip strength is also a useful prognostic tool for NAFLD [88,89]. The chair stand test measures the time required for a patient to stand up five times from a seated position without using the arms. This test is a reliable indicator of lower body muscle strength [90,91].

Skeletal muscle mass is measured to diagnose sarcopenia, and currently there are several methods for measuring skeletal muscle mass, including the dual energy X-ray absorptiometry (DEXA) method, computed tomography (CT) scan to measure skeletal muscle cross-sectionality at the level of the third lumbar vertebra, and the bioelectrical impedance analysis (BIA) method. For liver disease, CT scan which measures SMI, and the simple BIA method are often used. Recommendations for the CT method are based primarily on a 2017 multicenter retrospective study that examined wait-list mortality in patients based on pre-transplant sarcopenia [92]. This study established that the cutoff values for SMI at the level of L3 are <50 cm^2^/m^2^ for men and <39 cm^2^/m^2^ for women. The DXA method noninvasively measures a patient’s total body lean tissue mass or appendicular lean soft tissue mass [93]. DXA can easily provide reproducible estimates of accessory limb skeletal muscle mass in a matter of minutes [3]. The BIA method was developed based on the idea that tissues such as skeletal muscle, which are rich in water and electrolytes, offer less resistance to electric current than tissues such as bone, which are rich in lipids [94]. The BIA method does not directly measure muscle mass but derives an estimate of muscle mass from whole-body electrical conductivity [95]. BIA instruments are affordable, widely available, and portable, especially single-frequency instruments. The BIA method has been recognized as a viable means of assessing skeletal muscle mass in clinical studies with large sample sizes [96]. Since the DEXA and BIA methods are calculated from standard Japanese values, it is unclear whether they can be applied to patients with excessive obesity or edema such as in hepatic insufficiency, pleural effusion, or ascites. Although various modalities have been investigated to assess sarcopenia in liver disease, none have proven to be as clinically important a tool in screening for sarcopenia as the CT scan [97,98,99]. However, these tools are not commonly used in primary care due to the high cost of the equipment, lack of portability, and the need for highly trained personnel to use the equipment [100]. Recently, a new definition of sarcopenia combining muscle mass (e.g., grip strength) and physical activity (e.g., walking speed) in addition to muscle mass was published as a consensus in Europe, the United States, and Asia [101,102]. In addition, the Japanese Society of Hepatology has proposed its own sarcopenia diagnostic criteria [28]. In addition to muscle mass, skeletal muscle quality is also evaluated using CT and magnetic resonance imaging scans. Intramuscular adipose tissue content (IMAC), which is calculated by measuring the intensity of the multifidus muscle and dividing it by subcutaneous fat, reflects the adiposity of skeletal muscle, and magnetic resonance spectroscopy (MRS) has been used to evaluate the intramyocellular lipid content in muscle. Qualitative evaluation of skeletal muscle has been attempted by measuring intramyocellular lipid (IMCL) [46] in muscle by MRS.

Physical ability can be measured in a variety of ways by tests such as the gait speed test and the Timed-Up and Go (TUG) test. The gait velocity test requires the patient to walk 4 m. The simplicity of this test allows it to be performed in a wide range of medical settings [103,104]. The EWGSOP2 recommends a cutoff speed of 0.8 m/s or less as an indicator of severe sarcopenia [3]. During the TUG test, the patient is asked to rise from a chair, walk a distance of 3 m, turn around, return to the chair, and sit down again [105].

## 8. Treatment of Sarcopenia in NAFLD/NASH

The primary goal of treatment of NAFLD/NASH is weight loss by diet and exercise [106,107]. It is reported that 5% weight loss can improve the quality of life (QOL) as assessed by the chronic liver disease questionnaire (CLDQ). In addition, more than 7% weight loss is reported to improve liver steatosis in NASH [108], and more than 10% weight loss is reported to improve liver fibrosis [109]. Patients with high BMI may improve with caloric restriction, but conversely, weight loss may lead to further loss of muscle mass in patients with inadequate oral intake and increased muscle catabolism [110]. 

It is important to properly evaluate the state of sarcopenia and to appropriately combine aerobic exercise (such as walking) and anaerobic exercise (resistance exercise such as muscle training) in patients with NAFLD complicated with sarcopenia [111]. Exercise promotes the secretion of IGF-1 and decreases inflammatory cytokines such as IL-6, reactive oxygen species (ROS), and myostatin, thereby preventing sarcopenia [112,113]. In practice, exercise is not always possible for patients with physical mobility difficulties. Blood flow restriction (BFR) exercises are recommended as a training method that can achieve the same or better exercise results with very light weights compared with typical resistance exercises by restricting blood flow with a belt over a defined area [114].

Nutritional support such as providing BCAAs may also be useful in cases of cirrhosis. A retrospective study of cirrhotic patients with sarcopenia reported a significantly better prognosis in patients receiving BCAAs compared with those not receiving BCAAs [115]. In addition, vitamin D levels are considerably lower in patients with sarcopenia, irrespective of the presence of obesity [83,116]. It has been suggested that vitamin D deficiency is independently associated with the severity of NAFLD damage [117]. Vitamin D supplementation increases vitamin D receptor expression in skeletal muscle and improves sarcopenia [118].

Diet quantity and quality are vital for muscle health and are often overlooked [119], despite the consensus on adequate protein intake [120]. Because protein supplementation as a stand-alone intervention seems ineffective for improving muscle mass and strength [121], the ideal strategy involved the combination of exercise and adequate protein intake to mitigate sarcopenia in several contexts.

Recently, the efficacy of anabolic-androgenic steroids for sarcopenia has been reported [122]. Anabolic-androgenic steroids exhibit anabolic and androgenic effects via binding to androgen receptors and stimulate protein synthesis [123]. Whether drug treatment for sarcopenia can halt the progression of NAFLD/NASH may be an important clinical question for the future.

## 9. Conclusions

Sarcopenia in patients with NASH/NAFLD based on various factors such as insulin resistance and lipid factors may correlate with the progression of liver fibrosis and its prognosis. The condition of sarcopenia should be evaluated appropriately, and therapeutic interventions centered on diet and exercise therapy are necessary.

## Data Availability

The data that support the findings of this study are available from the corresponding author upon reasonable request.
